# New route for self-assembly of α-lactalbumin nanotubes and their use as templates to grow silver nanotubes

**DOI:** 10.1371/journal.pone.0175680

**Published:** 2017-04-12

**Authors:** Wei-Chun Fu, Mauricio A. Opazo, Sergio M. Acuña, Pedro G. Toledo

**Affiliations:** 1 Department of Food Engineering, University of Bío-Bío, Av. Andres Bello S/N, Chillán, Chile; 2 Department of Chemical Engineering and Laboratory of Surface Analysis, University of Concepción, Correo 3, Concepción, Chile; Russian Academy of Medical Sciences, RUSSIAN FEDERATION

## Abstract

Nanotubes are formed by self-assembly of α-lactalbumin milk protein following a different route than established for the hydrolysis which involves V8 enzyme, phosphate buffer and appropriate amounts of calcium at neutral pH. The resulting nanotubes are used as templates for the growth of conductive silver nanotubes. TEM, SEM-EDS, AFM and FTIR are used for characterization.

## Introduction

Molecular self-assembly occurs by spontaneous diffusion and specific association of molecules driven by non-covalent interactions such as hydrogen bonding, electrostatic interaction, hydrophobic packing, aromatic stacking and van der Waals forces. Although these forces are weak individually, when acting together they produce stable structures. A number of relatively recent studies used molecular entities as different as organic molecules, proteins, peptides, organometallic molecules, DNA and lipids, among several others, to produce novel nanoscale materials and devices intended for biological applications such as biosensors, probes, bionanowires and drug delivery systems, but also for nanotechnological applications such as micro/nanoelectronics, micro/nanofluidics and micro/nanoelectromechanical systems (for reviews see [1–10]). However, construction of useful nanoscale circuits with such devices remains a challenge due to the need for wiring between elements and electrical interfacing with macroscopic terminals [11,12].

Nanotubular structures self-assembled either from peptides or proteins are important structural elements that may serve as nanoscaffolds for the required conductive biologically-based nanotubes and nanowires [3,7–11,13,14]. Measuring electrical current through proteins, especially DNA, is, however, difficult and therefore there is still no firm conclusion about the mechanism of charge transport, if this is at all feasible. An alternative is to use protein-based nanotubes (PNTs) as templates for producing metallic nanotubes and nanowires with capabilities for electrical conduction. Thus, the mono-size condition, which is crucial in nanotechnology, would be assured [4–6,15–22].

Under certain conditions, it has been shown that partial hydrolysis of α-lactalbumin (α-La) protein of bovine whey by a *Bacillus licheniformis* protease (BLP) leads to tube-shaped nanostructures [3,23–30]. Ipsen and Otte recommend a protein concentration of 30 g/L to ensure formation of α-lactalbumin nanotubes (α-LaNTs) when the protease BLP is used and provided calcium is present [3]. In their case, nanotubes formed were ∼20 nm in diameter and several microns in length. The mechanisms of α-LaNT formation are fully described in the literature [3,28–31]. The presence of calcium in an appropriate amount is crucial for the self-assembly of α-LaNTs [32,33], which when achieved is easy to verify because the solution becomes a gel. The Ca^2+^ and α-La bond causes sharp structural changes, mainly in the tertiary structure with little or no change in the secondary structure of the protein. Calcium ions apparently form salt bridges between negatively charged carboxyl groups produced by hydrolysis [3], thereby increasing both persistence length and kinetics of self-assembly of α-LaNTs [27].

Here we use the α-La protein and a different route for the hydrolysis reaction to produce α-LaNTs. These nanotubes are then used as templates for the growth of conductive silver nanotubes.

## Experimental

### Preparation of protein nanotubes

For the production of α-lactalbumin nanotubes (α-LaNTs) we used the endoproteinase Glu-C from *Staphylococcus aureus* V8 protease (*S*.*aur*.V8). Glu-C selectively cleaves peptide bonds C-terminal to glutamic acid residues. Protein powder samples (α-Lactalbumin from bovine milk, Type III, calcium depleted, Sigma-Aldrich, USA) were diluted with 0.05 M phosphate buffer at pH 7.5 (A.C.E. Phosphate Buffer 50 mM, pH 7.5, bioWORLD, USA) to a final protein concentration of 3 g/L, the concentration of protein recommended by Ipsen and Otte when the endoprotease BLP is used and calcium is present [3]. For the hydrolysis we used instead the protease V8 (P2922–2.5 KU, Endoproteinase Glu-C, Type XVII-B, from *Staphylococcus aureus* strain V8, Sigma-Aldrich, USA) in a ratio proteinase/protein of 4% at pH 7.5. In almost all previous studies using BLP the Tris-HCl buffer at pH 7.5 was also used for the hydrolysis. The phosphate buffer used here has greater affinity for Ca^2+^ ions than does the Tris-HCl buffer, and thus a high concentration of Ca^2+^ was needed (Calcium chloride, Sigma-Aldrich, USA). To determine the optimal amount of calcium we studied a range of calcium to α-lactalbumin molar ratios (*R*) from 3 to 10 but only for values near 9 appeared a translucent gel indicating the formation of nanotubes. Here we used *R* = 9. Solutions were gently mixed at ambient temperature during 10 min and filtered immediately through a 0.1-micron filter (Millex^®^VV, Millipore Corp., Ireland). The filtrate was incubated for 90 minutes at 50°C in a thermo-regulated water bath (Julabo SW20, Julabo Labortechnik GmbH, Germany), then bottled and stored at 4°C for later examination. This incubation time was the minimum for the formation of a few nanotubes to facilitate analysis of images. For *R* = 9 we also study longer incubation times, up to 10 hours, giving rise to denser nanotube networks very difficult to characterize in images, although useful for improving the resolution of IR spectra.

### Nanotube characterisation methods

Filtrate samples after incubation were diluted 1:10 in 0.05 M phosphate buffer at pH 7.5, then equilibrated at room temperature for 10 minutes and finally examined with an electron microscope (JEOL/JEM 1200 EX II, Japan), operated at 120 kV, atomic force microscope (Dimension 3100, Veeco, USA) and FT-IR spectrometer (IRPrestige-21, Shimadzu, Japan).

TEM. Samples for transmission electron microscopy were prepared according to the following sequence: incubation, filtration and dilution 1:1 with a fixative (1.5% glutaraldehyde in 0.1 M sodium cacodylate buffer) and stored at 4°C.

AFM. The solution of α-LaNTs was diluted 1000 times in the same buffer used for preparation of the nanotubes. A sample of 20 μL of this solution was incubated at room temperature for 10 minutes on a glass surface previously immersed for 5 minutes in a solution of 1% DETA (Diethylenetriamine, Sigma-Aldrich, USA). The surface was washed with buffer to remove excess material and allowed to dry at room temperature. AFM was used in tapping mode.

FT-IR. An attenuated total reflectance (ATR) sampling accessory in combination with the FT-IR spectrometer provided the best means for determining the composition of the α-LaNTs. Infrared spectra were obtained over the range 1100–1800 cm^−1^ with a resolution of 1 cm^-1^ and 256 scans. The absorbance values were determined by mean of the IRsolution software (Shimadzu, Japan).

### Preparation and characterisation of silver nanowires

A 90 μL aliquot of a 2 mg/mL solution of α-LaNTs was added to 10 μL of a 10 mM AgNO_3_ solution boiling at 65°C. These conditions were applied before by Reches and Gazit to a different protein [18]. Citric acid was added as reducing agent to a final concentration of 0.038%. Unfortunately, the silver concentration proved too high and silver residues prevented a clear image of the nanowires. Therefore, we studied lower silver concentrations, that is, 7.5, 5 and 2.5 mM AgNO_3_ solution. The best TEM images were obtained with the 2.5 mM silver solution. In this way, three-layer coaxial nanowires were obtained with silver in the outer mantle and lumen of the α-LaNTs. When proteinase K was added to a solution of coaxial nanowires in a ratio of 100 μL/mL and the resultant solution was left for 1 hour at 37°C, the protein was degraded leaving the silver nanostructures exposed. Silver nanotubes were confirmed by images from TEM and AFM techniques described above. TEM images were obtained with Digital Micrograph 3.11.2 software (Gatan Inc., USA). AFM images were analysed using WSxM 5.0 software (Nanotec Electronica, Spain). The elemental composition of the metallic mantle of silver nanotubes was analysed by scanning electron microscopy (SU 3500, Hitachi, Japan) in combination with energy dispersive x-ray spectroscopy (Quantax, XFlash 6/100, Bruker, Germany). The samples were prepared by fixing the nanotubes to the microscope holder.

## Results and discussion

**Fig 1:**
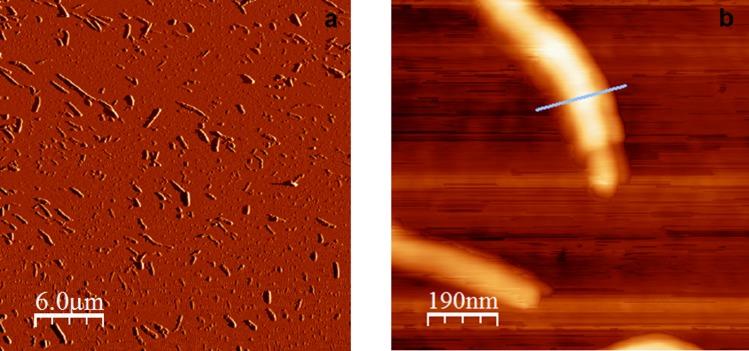
AFM micrographs of α-LaNTs self-assembled after partial α-La proteolysis at 50°C for 90 min. Before imaging, the sample was filtered and then highly diluted in 0.05 M phosphate buffer at pH 7.5. (a) Large area. (b) High resolution of a single nanotube.

Fig 1 shows high resolution AFM micrographs of α-LaNTs self-assembled after partial α-La proteolysis.

**Fig 2:**
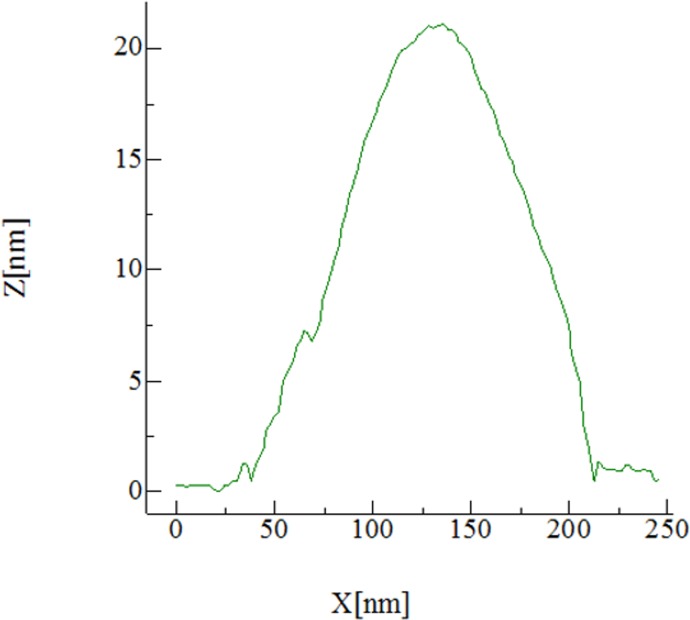
AFM height profile of the α-LaNT in Fig 1(B).

Fig 2 displays the AFM height profile of the nanotube in Fig 1(B). The height of the nanotube is close to 20 nm, a value that matches the diameter obtained using TEM (see below), and also that reported by Graveland-Bikker et al. [27]. However, the width of the nanotubes is significantly higher, close to 150 nm. This is not new; Graveland-Bikker obtained similar results for their nanotubes and concluded that the overestimation was due to the fat-tip effect [30]. The full-width half-maximum in Fig 2 is 90 nm and corresponds to the apparent width (*w*) of the nanotube measured with a large tip, the size of which is 50 nm according to the fabricant. According to Graveland-Bikker the width is related to the radius of the nanotube as $w=4\sqrt[{2}]{r_{tip}r_{nanotube}}$; applying this relationship, the nanotube in Fig 2 has a diameter of ∼20 nm as expected.

**Fig 3:**
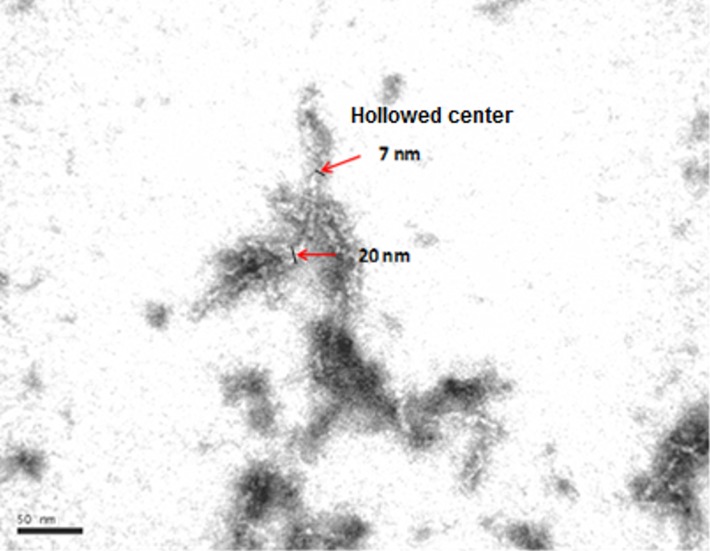
TEM micrograph of 3% (w/w) α-La incubated with 4% (w/w) V8 enzyme at 50°C (50 mM phosphate buffer, pH 7.5) at a calcium concentration of *R* = 9. Percolating nanotube network, walls and cavity of nanotubes are shown; arrows indicate external and inner nanotube diameters.

Fig 3 displays a transmission electron micrograph of the structure that is formed from α-La incubated with the V8 enzyme at 50°C at a calcium concentration of *R* = 9. A cloudy, viscous gel appeared when using very different values to 9; for low values we observed small but very tight clusters and for high values we observed large aggregates devoid of structure. Fig 3 shows a network structure of tubular elements apparently with a high coordination number; however, most of the linear elements are tangled rather than interconnected, and these latter elements are characterized as α-LaNTs. In the figure the walls and inner cavity of the nanotubes are clearly shown. The outer diameter of the nanotubes is generally 20 nm and the inner diameter 6.8 nm; this is in remarkable agreement with results from Graveland-Bikker et al. when using BLP enzyme at the same temperature [27].

The length of the nanotubes in Figs 1 and 3 varies from a few tens of nanometres to a few microns. Graveland-Bikker et al. suggest that the length of the nanotubes depends on both the time allocated to hydrolysis and the incubation time of the protein [31]; also, they deduced that nanotubes grow at a speed of 10 nm/min. For the nanotubes shown in Figs 1 and 3, grown at the same temperature and other conditions as Graveland-Bikker et al., the final length of the nanotubes should be 900 nm; however, the lengths obtained here range from 100 nm to over 3.5 microns in a few cases, with a mean value of 666 nm (SD 503 nm, N 203) as roughly predicted by Graveland-Bikker et al. but with a high dispersion which we attribute to the different enzyme used here. We are currently studying fine variations in incubation time to standardize the length of the nanotubes more accurately.

**Fig 4:**
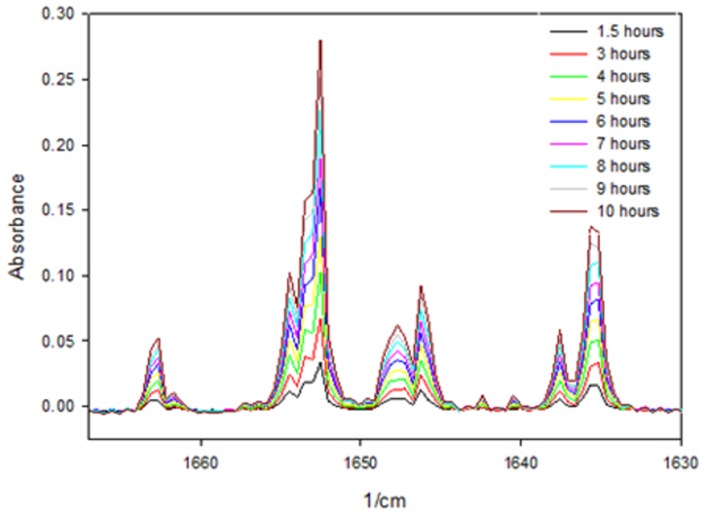
FTIR spectra in the region 1630–1670 cm^−1^ of α-La hydrolysed with enzyme V8 at increasing incubation times at 50°C.

We also conducted FTIR analysis for better evaluation of the structural changes in the α-La as the enzymatic reaction advanced with the enzyme V8. Fig 4 shows the infrared spectra in the region 1610–1700 cm^−1^ corresponding to Amide I, the major band of a typical protein. The Amide I band is mainly associated with the C = O stretching vibration (70–85%) and significantly less with the C–N groups (10–20%). The exact band position is determined by the backbone conformation and the hydrogen bonding pattern. Here, Fig 4 shows that the Amide I band increases with the hydrolysis, particularly the peak at 1652 cm^−1^ associated with C = O groups. We observed that longer incubation times led to more extensive hydrolysis of the α-La and to a larger amount of peptides in the sample, thus increasing the number and intensity of the C = O stretching.

**Fig 5:**
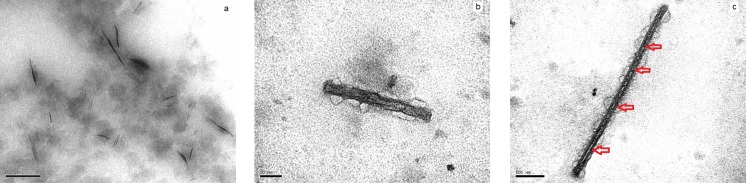
TEM micrographs of α-LaNTs coated with silver. (a) Several nanotubes. (b) A single nanotube. (c) A silver nanotube with discontinuous silver in its lumen, the small red arrows point to silver free pockets.

To facilitate observation of the metallized nanotubes formed, the solutions were diluted 1 : 10 in 0.05 M phosphate buffer at pH 7.5. Fig 5 shows micrographs of α-LaNTs coated with silver which has been reduced over the mantle of the nanotubes. Therefore, nanotubes are observed more clearly than in their native state, free of silver, as seen in Fig 5(A). Fig 5(B) shows that silver has been deposited continuously on the mantle of the nanotube. Globular translucent material around the nanotube corresponds to small excesses of both fixative and stain solution. To remove the protein portion of the silver-metallized α-LaNTs, and to try to access their interior, enzymatic degradation was performed using proteinase K at a concentration of 100 μg/mL for 1 h at 37°C [7,18]. Proteinase K is a protease which acts on the weaker bonds of the protein, reducing it to its own primary structure devoid of protein functionality. Fig 5(C) shows the results of degradation of an α-LaNT covered with silver. In this micrograph we observed an elongated structure about 20 nm in diameter, equivalent to the average outer diameter of α-LaNTs. Inside the nanotube, axially, there are small pockets free of silver. When sizing these pockets, we found that their width is close to 7 nm, exactly the same value of the diameter of the cavities of α-LaNTs. According to the observed structures, silver tended to be deposited in a very thin layer, preferably on the outer mantle of α-LaNTs. The presence of elemental silver was verified in EDS spectra, one of which is shown in S1 Fig. The 7 nm pockets correspond to space inside the nanotube discontinuously filled with silver. We speculate that the silver pockets could be connected continuously with the silver mantle, possibly due to partial degradation of the α-LaNTs by the citric acid. If so, the silver nanotubes would be more resistant. This will be evaluated through ongoing mechanical resistance tests. The possibility that the discontinuous material in the lumen of the α-LaNTs were non-degraded protein is completely discarded simply because the lumen prior to exposition to silver is a hollow core. Silver is stabilized on the outer mantle of the α-LaNTs mainly by electrostatic forces. Thus, we have fabricated silver nanotubes using α-LaNTs as templates. Currently, we are working to find the optimum concentration of silver nitrate that allows nanotubes filled with silver continuously.

## Conclusions

Nanotubes were formed by self-assembly of α-lactalbumin milk protein following a different route for the hydrolysis which involves V8 enzyme, phosphate buffer and the presence of appropriate amounts of calcium at neutral pH. All necessary components are readily available. The phosphate buffer had greater affinity for calcium ions than buffers used before, and thus a high concentration of calcium ions was needed. The optimal amount measured as calcium to α-lactalbumin molar ratio was 9, only close to this value appeared a translucent gel indicating the formation of nanotubes. The resulting α-lactalbumin nanotubes were geometrically identical to those obtained previously with different combinations of enzyme, buffer and calcium ratios, nanotubes were hollow with outer diameter around 20 nm, inner diameter around 7 nm, and long persistence length on the order of micrometers. The α-lactalbumin nanotubes were used as templates for the manufacturing of conductive silver nanotubes. Uniform silver nanotubes with 20 nm in diameter were obtained after degrading the α-lactalbumin nanotubes with protease K. The small internal cavity of the alpha-lactalbumin nanotubes was only partially filled with silver; fine variations in the amount of silver should lead to silver nanowires. Applications for α-lactalbumin nanotubes have been identified previously, for example, for viscosifying, gelation and encapsulation purposes. In addition, silver nanotubes and nanowires, by their unique uniform size and properties may be used for the electrical interfacing between nanoscale devices.

## Supporting information

S1 Fig EDS spectrum of silver nanowires. The strongest signal occurs in the Ag^0^ region confirming the presence of elemental silver. Also present O, Na and P.
(TIF)
