# Supplementary figures and images for: New route for self-assembly of α-lactalbumin nanotubes and their use as templates to grow silver nanotubes
Source: PLoS One. 2017 Apr 12;12(4):e0175680. doi: 10.1371/journal.pone.0175680 (PMC5389836; doi:10.1371/journal.pone.0175680)

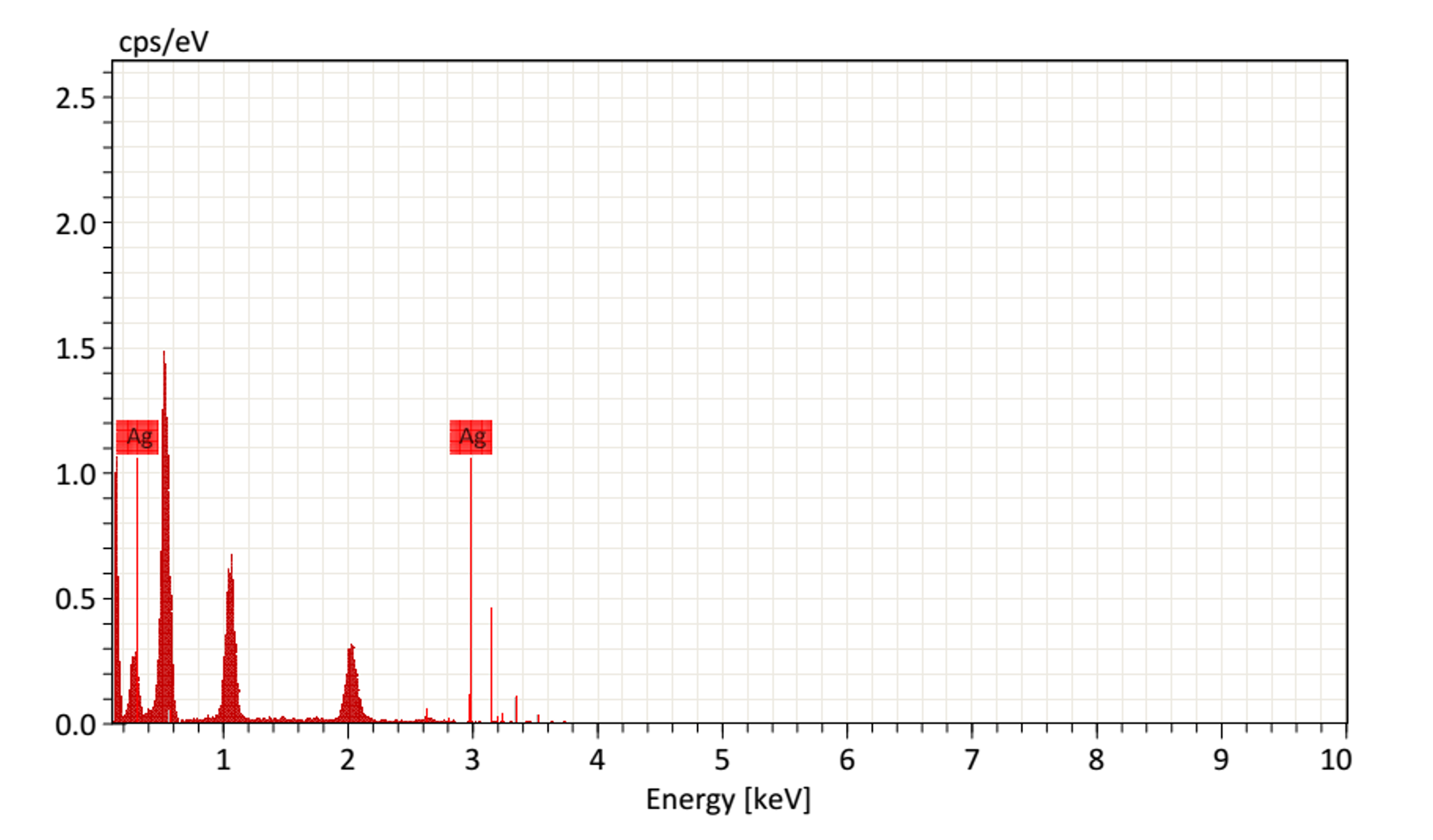

Supplement: S1 Fig — The strongest signal occurs in the Ag0 region confirming the presence of elemental silver. Also present O, Na and P. (TIF) [file pone.0175680.s001.tif]
